# The Impact of Noise Anxiety on Behavior and Welfare of Horses from UK and US Owner’s Perspective

**DOI:** 10.3390/ani12101319

**Published:** 2022-05-21

**Authors:** Maria Giorgia Riva, Francesca Dai, Mirja Huhtinen, Michela Minero, Sara Barbieri, Emanuela Dalla Costa

**Affiliations:** 1 Department of Veterinary Medicine and Animal Science, University of Milan, 26900 Lodi, Italy; maria.riva@unimi.it (M.G.R.); michela.minero@unimi.it (M.M.); sara.barbieri@unimi.it (S.B.); 2 Il Rifugio degli Asinelli ONLUS, 13884 Sala Biellese, Italy; francesca.dai@ilrifugiodegliasinelli.org; 3 Orion Corporation, Orion Pharma, R&D, 02200 Espoo, Finland; mirja.huhtinen@orionpharma.com

**Keywords:** noise anxiety, horse welfare, management strategies, fear behavior

## Abstract

**Simple Summary:**

Being a prey species, horses evolved to rapidly react to potential danger and loud noises may be perceived as such. Therefore, they can exhibit several anxiety behaviors during noisy events, including sweating, trembling and escape attempts, which may cause severe accidents for the horse and the rider/handler. Since noise anxiety can significantly impact on horse welfare, the aim of the present study was to investigate owners’ perception of noise anxiety severity in their horses, their management strategies and perceived efficacy. In a survey, 409 out of 1836 owners reported that their horse shows unusual behavior during a noise event. Among those, two noise anxiety clusters could be identified: very and slightly anxious horses. Very anxious horses were reported to have higher frequency of all anxiety behaviors and higher frequency of signs of noise reactivity; furthermore, their anxiety did not improve with time. The most frequently used management strategies were providing hay throughout the night or turning out or moving horses to a paddock; though, most of these techniques were reported to be effective only in the slightly anxious subjects. Our results confirmed that noise anxiety is a widespread behavioral problem (22% of our respondents reported that their horse had ever shown any unusual behavior in relation to loud noises) that can lead to negative welfare consequences for horses.

**Abstract:**

Noise anxiety is an over-reaction to loud noises commonly detected among pets and can greatly impact on their welfare and on their management. When exposed to noisy events, horses can show intense escape attempts, which may cause severe accidents for the horse and the rider/handler. The aim of the present study was to investigate, through a web survey, UK and US owners’ perception of noise anxiety severity in their horses, their management strategies and perceived efficacy. The questionnaire was shared via social networking and advertised as “What is your horse afraid of?”. Over a total of 1836 questionnaires filled out; 409 owners reported that their horse has shown unusual behavior during a noise event. A two-step cluster analysis identified two groups: very anxious (VA) and slightly anxious (SA). VA horses were reported to have higher frequency of anxiety behaviors; higher frequency of signs of noise reactivity; and their anxiety did not improve with time. The most used management strategies consisted in providing hay throughout the night, turning in/out their horse or moving it to a paddock. A binomial logistic regression identified that horses that have reported injuries during noise events were more likely to be clustered as VA (OR = 0.24, 95% CI: 0.08–0.76); while providing hay throughout the night was more likely to be very effective management strategy in SA horses (OR = 0.41, 95% CI: 0.16–1.01). Our results confirmed that noise anxiety is a growing behavioral problem that can lead to important welfare concerns for horses. New management strategies, including the use of medicinal products, should be considered to reduce behavioral and physiological signs and help horses to cope with noisy events.

## 1. Introduction

Fear can be defined as a reaction to the perception of actual danger, while anxiety as the reaction to a potential danger that threatens the individual [1]. Reaction to fear elicits behavioral and physiological modifications [2], such as active defense (attack, menace), active flight (hiding, escape) and passive avoidance (freezing) [3], heart rate and heart rate variability variations [4,5,6,7,8,9] and rising in cortisol concentration [10,11,12]. Horses can exhibit several behaviors as fear reaction, including sweating, trembling and escape attempts, which may be particularly dangerous, causing severe accidents for the horse and the rider/handler [4,13,14,15].

Noise can be described as unwanted sound, eliciting disagreeable feelings in the listener [16]. The response of animals to a certain noise, is determined not only by sound intensity, loudness, frequency, duration and pattern, but also by the animal’s previous experience and the acoustic stimulus predictability [17]. Exposure to unknown sounds could elicit stress in animals [16,18,19,20,21]. For instance, aircraft and helicopter noise has been reported to affect feed intake, growth and production rates in domestic animals [22,23,24]. Compared with chronic background or repetitive noise, sudden high intensity unpredictable noises cause greater fear reactions in mammals, including startle response, freezing and fleeing from the sound source [16].

“Noise aversion” or “noise anxiety” occurs when an animal over-reacts to loud noises [25,26,27]; this phenomenon is well studied in pet dogs. Reported behaviors of dogs experiencing noise anxiety comprise trembling, shaking, salivating, hiding, panting, pacing, restlessness, inappropriate elimination, soliciting human attention, escaping confinement, hiding, destruction, loss of appetite or barking [27,28,29]. Data suggest that the prevalence of noise anxiety in dogs could reach 49% [26], representing indeed a serious welfare problem. Thunderstorms, fireworks and gunshots are the most commonly reported anxiety-eliciting sounds [25,26], a common characteristic is to be loud explosive noise. Other sound reported to cause anxiety problems are loud sounds on TV, cars backfiring and hoovers [26]. Evidence suggest that dogs commonly generalize fear of one to other noises [26].

Being a prey species, sensory systems of horses evolved to enable a prompt detection of potential danger, throughout a combination of visual, auditory and olfactory cues [14,30]. Compared to visual and olfactory stimuli, unexpected unknown noises seem to elicit rapid flight reactions more frequently [14,31].

Among other noise events, fireworks are the commonest sound causing a fear response [26]. Fireworks may elicit fear reactions in animals, including horses, due to unpredictable, intermittent and high-intensity sounds, with light flashes, odors and changes in barometric pressure [32,33,34]. Anxiety behaviors related to fireworks are very commonly detected among pets. In a survey conducted in New Zealand, 74% of owners reported their dog or cat to experience noise anxiety caused by fireworks [35]. The reported prevalence of noise anxiety in dogs ranges between 23% and 52% [26,29,36,37,38]. Breed, genetics, age, health conditions and environment are recognized risk factors for noise anxiety in dogs [26,36,37,38]. Full recovery from fireworks-related fear is reported to happen the next morning in 75% of dogs, while some subjects may even display milder symptoms for days or weeks after [38]. This clearly represents an animal welfare problem. Compared to dogs, cats are more likely to exhibit behaviors such as hiding or cowering in response to frightful noise stimulation, which owners often fail to correctly interpret as sign of anxiety [29]. Therefore, cat’s noise anxiety caused by fireworks is less frequently reported by owners [29,39].

While a number of publications describe the incidence of fear behaviors in response to fireworks in dogs and cats [26,29,32,40], only few studies are published on horses. Gronqvist and colleagues [33] reported that 79% of horses in New Zealand were rated as anxious or very anxious around fireworks by their owners, and, as a consequence, 26% obtained injuries during fireworks. Young and colleagues reported that horses, listening to the sound of fireworks played from compact disk, had higher cortisol levels, indicative of anxiety, than horses reacting to sound from coat clippers or being kept in social isolation [41].

Moving the horse to a paddock away from the fireworks was reported by Gronqvist and colleagues [33] to be the most common management strategy adopted to mitigate this problem; the paper highlights, however, that 37% (374 out of 779) of the respondents reported that this management strategy was ineffective. In dogs and cats, several therapies are described to manage and eventually even overcome noise anxiety and fear. Generally, the condition can be treated using a system of desensitization and counter-conditioning [42], but this may take several weeks. As an immediate solution during the fireworks event, medications such as sedatives and anxiolytics can be used to manage the animal behavior [32,34,42,43,44]. To reduce animal’s reactivity by sedation, Acepromazine has been used for decades, but it can often give disappointing results and cause undesirable side-effects [45,46]. Therefore, benzodiazepines, such as diazepam, or serotonin reuptake inhibitors are now recommended to provide an immediate anxiolytic effect during the anxiogenic event [47]. Diazepam is commonly used [48] and its reported efficacy is generally good [49], however adverse side effects include aggression, sedation and ataxia [45,49]. Moreover, undesirable effects of benzodiazepines includes a reduction of conditioned responses and memory deficits, thus potentially compromising behavioral modification [45]. The use of Detomidine for alleviation of anxiety during noisy events have been tested both in dogs [50] and in horses [34]. The studies suggest that this drug has a good efficacy in reducing anxiety-related behaviors, as reported by owners. While in horses no side-effects have been reported, in dogs observed side-effects included emesis, gastroenteritis, periorbital edema, drowsiness and sedation. The use of Detomidine for this purpose in horses, indeed, is off-label and more studies are needed to confirm its efficacy and safety [34].

To the authors knowledge, no data on horse anxiety around other noises are published. While owners can easily recognize noise anxiety in pet since they share the same house, noise anxiety in horses may not have taken very seriously, probably due to the fact that it is not obvious for owners if they are not around during the noise event [51].

Housing condition has an impact on horse reactivity. Single box housing is recognized as a risk factor for the development undesired behaviors, such as stereotypies, aggressiveness, hyperreactivity or unresponsiveness [52,53,54,55,56,57,58,59]. Lesimple and colleagues [60] demonstrated that horses housed in single boxes exhibit more strong reactions to unknown stimuli and are more prone to express “high locomotory components”, thus increasing risks of accidents. Since noise anxiety can significantly impact horse welfare, the aim of the present study was to investigate, through a web survey, UK and US owners’ perception of noise anxiety severity in their horses, the management strategies adopted to reduce anxiety behaviors, and their perceived efficacy.

## 2. Materials and Methods

### 2.1. Ethic Statement

Horse owners entered the study on a voluntary basis, they were informed about the objectives of the project. The study did not focus on human subjects or human data, no sensitive data were collected, it was not possible to identify the participants from the raw research data. Ethical consent was deemed unnecessary as no personal details of the participants were recorded and the EU Regulation No. 2016/679 does not apply.

### 2.2. Online Questionnaire

An online questionnaire was created using the commercial survey software Webropol Survey Analytics (Webropol Ltd., Helsinki, Finland). The defined target population was horse owners located in United Kingdom and United States. The questionnaire and its advertisement were written in English, for this reason and because of their renowned equestrian tradition the survey was conducted in these two Countries. The estimated horse population in Britain stands at 847,000 base on BETA National Equestrian Survey 2019 data [61], and American one at 3.8 million in 2017 [62]. Invitations to contribute to the survey were shared via social networking sites (Facebook, Meta Inc., Menlo Park, CA, USA) with the advertisement “What is your horse afraid of?” on a horse picture. By clicking on the picture, respondents had access the questionnaire; separate links for UK and US were created. The questionnaire was open for one month (April 2018), and it could be completed anonymously. Participants were required to be over the age of 18, therefore Facebook’s customers of age were selected, but no IP address or any other personal data were recorded. The questionnaire was organized in two parts, the first one included three multiple-choice questions (Table S1). Only the respondent who answered “yes” to the question “Has your horse ever shown any unusual behavior (e.g., restlessness, sweating, decreased appetite) during noisy events such as firework displays?” could access the second part of the questionnaire. The second part of the questionnaire consisted of 16 multiple-choice questions reported in Table S1. Closed-ended questions were chosen as it is reported that customers are more likely to respond and answers are easier to compare. The questions were formulated to be as consistent as possible with previous publication, Gronqvist’s study [33]. For three questions (e.g., Which of the following behaviors has your horse shown during noisy events?; In your opinion, which are your horse’s three most alarming behaviors related to noise reactivity?; Please indicate if you have tried the following management strategies for your horse during noisy events such as firework displays in previous years and how effective the management strategy was) the option “other (specify)” was provided, however, the collected answers were sometimes irrelevant (not related to the question) and sometimes difficult to interpret; for this reason they were excluded from statistical analysis.

### 2.3. Statistical Analysis

Data were analyzed with SPSS statistical package (IBM SPSS Statistic 27). For statistical analysis purposes dichotomous variables (Yes/No) were created for the replies to the questions “In your opinion, which are your horse’s three most alarming behaviors related to noise reactivity?”. Descriptive statistics including relative proportions were calculated. Anxiety behavior score was calculated adding the frequency (Never = 0; Sometimes = 1; Always = 2) of anxiety behaviors reported by horse owners. A two-step cluster analysis with automatic determination of the number of clusters was performed on the anxiety behavior score to identify groups of horses that were similar to each other for the considered variables. The association between horses’ anxiety level identified with cluster analysis and other independent variables was investigated using binomial logistic regression, reporting odds ratios with 95% CIs. Differences were considered to be statistically significant if *p* < 0.05.

## 3. Results and Discussion

A total of 1836 questionnaires were correctly filled out, the majority coming from respondents living in the United Kingdom (*n* = 1220, 66%); this could be due to a diverse use of social media. Main information about owners and horse population are showed by Table S2. Most of the respondents owned one horse (*n* = 928, 50%), followed by respondents owning two or more horses (*n* = 846, 46%); only 51 (less than 3%) respondents did not own any horse. These last ones were considered in the survey because they actually managed their horses, even if without real possession (lease or half lease contracts). Considering the experience with horses, 114 respondents (6%) reported to have less than 2 years of experience with horses, 236 (13%) had between 2 and 5 years, while most of the respondent had from 6 to 20 (*n* = 754, 41%) and over 20 years of experience (*n* = 726, 40%). As shown also by surveys carried out in Sweden and Finland, by owning one or more horses there is a higher probability to be involved in noise anxiety episodes, and therefore be attracted to this kind of survey on the internet [63]. Likewise, the longer experience with horses the respondents had, the higher the probability to have ever observed a noise anxiety-related behavior. Only 409 owners (22%) reported that their horse had ever shown any unusual behavior and, therefore, completed the second part of the questionnaire. This result is lower than what was found in New Zealand by Gronqvist and colleagues [33], where 79% of participants reported their horse to be anxious or very anxious during fireworks display. Furthermore, Lindstedt’s survey reported higher percentages: 30% of Finnish participants and 55% of Swedish ones answered that their horse has shown signs of noise anxiety during loud noise events [63]. A subset of 376 informative answers in our survey were selected for further statistical analysis: questionnaires where owners left blank or answered “don’t know” for more than half of the questions were excluded (*n* = 33). A two-step cluster analysis identified two clusters based on the anxiety score: forty-eight percent (*n* = 181) of the horses were assigned to the first cluster and fifty-two percent (*n* = 195) to the second. Horses in the second cluster showed a higher frequency of all these anxiety behaviors compared to horse in the first one. Therefore, cluster 1 was labelled as “slightly anxious” (SA) and cluster 2 as “very anxious” (VA).

### 3.1. Horse and Stable Characteristics

In the SA cluster, most of the horses were adults: only one horse was younger than 1 year old, a few from 1 to 5 years old (12%), the majority between 6 and 15 years old (59%) or over 15 years (29%). Horses clustered as VA were characterized by a similar age distribution: no horse was younger than 1, 6% was between 1 and 5 years, 60% of the horses ranged from 6 to 15 years old and 34% was older than 15 years. The VA cluster comprised a higher number of geldings (65%) and a lower number of mares (35%) compared to the SA, respectively, 54% and 46%. A lot of different and specific breeds were reported; therefore, they were grouped in three major categories (hotbloods, warmbloods and coldbloods) to simplify the data analysis. The majority of respondents reported to own warmbloods (SA 52%; VA 49%), followed by coldbloods (28% and 26%, respectively) and hotbloods (SA 17%; VA 22%). The most common use for these horses was riding (show jumping, dressage and eventing), followed by leisure and western riding activities. Only a few horses were retired, or used for breeding or driving. The majority of the survey participants reported to keep their horses in a rural (surrounded by farms) or semi-rural (adjacent to an urban area) location. A greater proportion of participants reported checking their animals during noisy events (SA 81%; VA 92%).

### 3.2. Anxiety Behavior

Noise anxiety was displayed through a variety of anxiety behaviors (e.g., fence/box walking, running, appetite loss, diarrhea, breaking fence, weaving, bucking, sweating, fever, trembling and vocalization). The frequency of different behaviors showed during noisy events in each cluster is reported in Table 1. Most of the anxiety behaviors were reported to be exhibited with “sometimes” frequency. This was particularly true for the VA cluster, while SA horses’ owners have rarely observed behaviors such as diarrhea, fever, or breaking fences. These results already highlight the different level of arousal between the two categories. The higher anxiety level in VA horses causes more severe consequences both from physiological and behavioral perspective.

Fence/box walking and running were the most observed activities, immediately followed by sweating. Running was also the most noted sign found in Gronqvist and colleagues’ study [33], whereas in Lindstedt’s survey general anxiousness was the most reported sign, followed by decrease appetite, diarrhea or increased defecation, running in the box and sweating [63]. The odds of horses showing walking or running behaviors with a “sometimes” frequency was quite similar between the two clusters. However, 28% and 22% of VA horses’ owners reported that their animals “always”, respectively, walk or run during a noisy event, while 29% and 27% of SA horses have “never” shown, respectively, walking or running behaviors. A peak of 150 VA horses (77%) and 107 SA ones (59%) sweated with a “sometimes” frequency. Conversely, 39% of SA horses and only 4% of VA horses have “never” sweated during noisy events. A few respondents reported gastrointestinal symptoms: many of the horses classified as VA have “sometimes” shown appetite loss (60%) and/or diarrhea (54%), whereas SA subjects were quite unlikely to experience these conditions. Even though fever was a symptom that showed up rarely in the survey, 3 participants have “always” observed an increase in body temperature. VA horses appeared to be more likely to break fences (40%) than SA ones (13%), even if the vast majority of the owners did not report this behavior at all (*n* = 265, 70% of the respondents). VA horses were also quite likely to rear or buck: 61% of the owners answered that their animals have “sometimes” shown these behaviors and 12% that they have “always” behaved in this way. A great proportion of the participants (55%) did not observe the occurrence of weaving behavior, but, respectively, 46 (25%) and 87 (45%) SA and VA horses’ owners reported their horses having “sometimes” shown this symptom or other stereotypic behaviors. Even trembling and vocalization were reported as more usual in VA horses than in SA ones. Many of these behaviors, such as fence walking, weaving and other stereotypic behaviors, are reported in the list drawn up by Young and others [41] to identify a horse’s stress level. The matching between the behaviors listed by Young and those reported by the questionnaire’s respondents may indicate that fireworks cause medium to high levels of distress in horses and therefore they should be addressed as a cause of welfare issues. 

**Figure 1:**
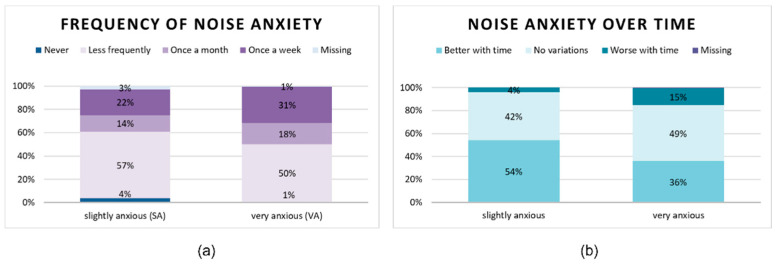
Graphs showing for each cluster: (**a**) the frequency of noise anxiety and (**b**) evolution of noise anxiety over time.

Most of the owners did not know when the anxious reaction to noisy events had appeared for the first time (SA 48%; VA 35%). Regardless, when the first unusual behavior had been observed by the owners, the majority of them reported that to have happened in a range between 4 and 9 years old (SA 23%; VA 31%). Only 10 horses in each cluster (SA 6%, VA 5%) had shown their first anxious response before turning 1 year old. When the frequency of anxiety behavior is considered (Figure 1) in both groups, approximately half of the respondents reported their horses to have anxious episodes less than once a month (SA 57%; VA 50%). Nevertheless, in the VA cluster 35 (18%) horses showed signs of noise reactivity one a month and 61 (31%) even once a week. The vast majority of the SA horses’ owners noticed that this behavior improved with time (54%) or did not change (42%). VA horses tended either not to change in their reactivity (49%) or to improve with time (36%), while others got worse (15%). Even in the Nordic answers, there were indications that the more anxious horses got worse with age [46]. Fireworks are intermittent and high-intensity noisy events and therefore totally unpredictable for a horse, making the habituation process hardly possible. Thus, particularly very anxious subjects might have no improvements or even get worse with age, which has already been proven to be common in dogs [36,37,38].

The majority of respondents reported their horses limit anxious behaviors to the duration of the noisy event (SA 80%; VA 49%). In some cases, especially with very anxious subjects, anxious behaviors lasted up to 2 h after the noisy event ended (35%) or even until the next day or longer (14%). In the Nordic questionnaire, owners that reported anxious signs lasting longer than the end of the fireworks had around four times higher risk of owning a horse with severe noise anxiety [46]. Literature reports similar findings in dogs, whose signs may last even for a few weeks [39].

Based on this survey, VA horses are more likely to injure themselves during noisy events (26%) than SA ones (5%). Gronqvist and colleagues’ survey yielded an even larger proportion (a quarter) of participants reporting injuries associated with fireworks, and multiple different types of injuries were described from minor cuts and sprains to broken limbs [33]. Therefore, noise anxiety in horses can be considered as a significant welfare issue, both due to the unnecessary fear and distress it causes to the animal, and due to its physical consequences, namely wounds and injuries. Another thing worth mentioning is the risk of potentially dangerous accidents for the rider/handler [13,14,29].

### 3.3. Most Alarming Behaviors

**Figure 2:**
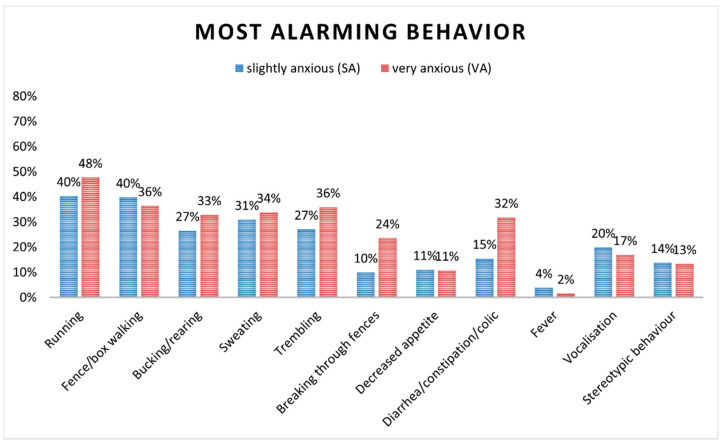
Graph showing, for each cluster, the reported percentages of most alarming behaviors perceived by horse owners.

Owners’ opinions on the most alarming behaviors are presented in Figure 2. A greater proportion of participants in both clusters stated to be worried about behaviors that lead to “excessive activity”, such as running (SA 40%, VA 48%), fence/box-stall walking (SA 40%, VA 36%) and bucking/rearing (SA 27%, VA 33%). Additionally, sweating and trembling were regarded as alarming behaviors in both clusters. VA horses’ owners reported breaking fences and gastrointestinal symptoms as most alarming behaviors more often than SA horses’ ones. When asked to rate how anxious their horses were in these circumstances, only 12 owners (SA = 11, 6%; VA = 1, 1%) described them as “not anxious at all”.

### 3.4. Horse Management during Noisy Events

The participants were asked to describe how they used to deal with their horses’ noise reactivity and how effective the different management approaches had been. The vast majority of the strategies that had been used by the owners were reported to be more effective in the slightly anxious subjects than in the very anxious cluster. A greater proportion of the participants reported that, during fireworks, they had provided hay throughout the night, turned in/turned out their horse or moved it to a paddock away from the event. The last two were also reported to be the most popular strategies by Gronqvist and colleagues [33], possibly because they are easier management solutions than looking for areas off the properties or asking veterinarians for prescription medicines. Moreover, Lindstedt [63] showed quite similar results: keeping the horse in the stable, playing music/radio and providing hay throughout the night were reported to be the most used management techniques by Finnish and Swedish owners. Regardless, in the same study, none of these protocols were considered effective by all the owners.

**Figure 3:**
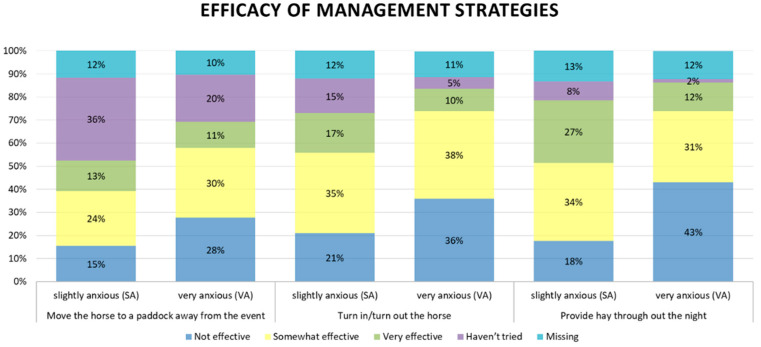
Graph reporting the perceived efficacy of different management strategies by horse owners of the two clusters.

Different perceived efficacy levels of the three main management techniques are shown in Figure 3. Providing hay during nighttime proved to be quite effective in the SA group; conversely, most of the VA horses did not show any improvement. Moving the horse to a paddock or turning it in/out tended to be assessed as “somewhat effective” in both clusters; however, VA horse owners reported moving the horse to a paddock as “not effective” more frequently than SA horse owners. Similarly, owners in the SA group reported turning the horse in/out to be “very effective” more frequently than the VA group, while VA perceived this procedure as “not effective” more frequently (*p* = 0.002). Only few respondents answered that they had sedated the horse (using prescription medicines), used over-the-counter products, used ear plugs/covers, moved it off the property, covered the windows or played music.

When asked about future management strategies there were no differences between the owners of horses belonging to the different clusters. Most of the participants planned to provide hay throughout the night (SA 52%; VA 47%), turn in/turn out the horse (SA 38%; VA 45%) and play music or radio in the stable (SA 22%; VA 30%). Only five (3%) SA horses’ owners would use sedation, while a few more (7%) VA horses’ owners planned to sedate their horse during the upcoming noisy events.

The survey ended with a specific question on owners’ interest in using new medicinal products for controlling noise anxiety. Most VA horses’ owners stated to be quite interested, 23% and 39% of them answered “yes” or “likely yes”, respectively. While SA horses’ owners were less inclined to adopt this kind of management strategy, only 8% of them answered “yes”, and 28% “likely yes”. Paste and powder/granules were the preferred medication form (SA 85%; VA = 71%). Only 14% of SA horses’ owners and 29% of VA ones chose oral liquid/solution, oromucosal gel or tablets. Similar results were yielded from Swedish and Finnish horse owners: the ones who reported severe noise anxiety in their horse also expressed their potential interest in the use of prescription drugs [63]. Dangerous and risky behaviors, longer duration and difficult management made VA horses’ owners concerned about noise anxiety. As reported, these horses are more likely to injure themselves and literature reports a close link between anxious behaviors and physiological signs of distress [2,3,4,5,6,7,8,9]. Therefore, noise anxiety can be considered a health issue, both because the risk of wounds and cuts and because of the stressful condition that may lead to severe systemic disease. Consequently, it is crucial that the owners consult their veterinarian to find the best management strategy, including medicinal products when necessary. Pharmacological treatment options are available for dogs with noise anxiety. Dexmedetomidine [50] and imepitoin [64] have an anxiolytic effect, especially with noise-related anxiety. However, there are no registered medicines for noise anxiety in horses, thus all drugs are used off-label. 

A recent study [34] investigated the use of detomidine (Domosedan vet 7.6 mg/mL oromucosal gel—Orion Corporation, Espoo, Finland) in acute anxiety episodes in horses. The results suggested that detomidine could be effective in alleviating acute fear and anxiety triggered by firework-related noise, and no significant adverse effects were observed. However, this was a pilot study with some limitations (small sample size, possible uneven fireworks intensity and a horse selection based mainly on owners’ reports), thus further research is still needed. 

### 3.5. Logistic Regression

A binomial logistic regression was performed to ascertain the effects of the independent variables (e.g., breed and sex of the horse, location of the stable, noise anxiety frequency, duration and changes over time, age horse start showing noise anxiety signs, most alarming behaviors considered by the owner, injuries and effectiveness of the tree most used management techniques) on the likelihood to be part of the VA group. The model included the independent variables listed above except country and experience of the respondent (which were only included in the study for descriptive purposes), and horse characteristics such as age at the moment of the questionnaire and main purpose (on which the authors found no published data about how they are commonly associated with noise reactivity in this species). The model was statistically significant, χ^2^ (39) = 152.150, *p* < 0.001, explaining 51.4% (Nagelkerke R2) of the variance in the anxiety level and correctly classified 81.5% of cases. Table 2 summarizes the results of logistic regression. Horses that have reported injuries during noise events (OR = 0.24, 95% CI: 0.08–0.76) were more likely to be clustered as VA. Horse owners who reported to be mostly alarmed by behaviors such as breaking through fences (OR = 0.17, 95% CI: 0.06–0.48), bucking/rearing (OR = 0.39, 95% CI: 0.18–0.87), gastrointestinal signs (OR = 0.23, 95% CI: 0.10–0.56), running (OR = 0.39, 95% CI: 0.18–0.83) or weaving (OR = 0.34, 95% CI: 0.12–0.92) were more likely to have a horse in the VA cluster. Gronqvist and colleagues [33] highlighted a possible association between certain alarming behaviors, such as breaking through fences, and an increase risk of injuries and lacerations. This may explain the higher concern of the VA horses’ owners for all these “intensive activity” related behaviors: breaking through fences, bucking/rearing and running. In addition, horses, as a species, are highly susceptible to suffering anxiety-related problems [65], but, as mentioned by Stuijfzand and colleagues [66], also humans with higher anxiety levels tend to show hyperreactivity even to moderate or ambiguous threatening stimuli. Consequently, these hyperreactive subjects are more prone to manifest not only behavioral alterations but also physiological changes due to the chronic stress response [67]. Anxiety activates hormonal and neuronal pathways causing changes such as fluctuations in epinephrine and cortisol levels; as a consequence the heart rate increases and the blood flow is altered, leading to a reduction in gastrointestinal activity among others [68,69]. VA horses’ owners may be particularly worried about gastrointestinal signs because in their animals these physiological changes turn into pathologies, such as diarrhea or even colic, more frequently than in less anxious subjects. Furthermore, this hyperreactivity status decreases the level of well-being of the horse, its ability to cope environmental changes while increasing negative emotional states [65]. Thereby, these high levels of frustration may justify the development of stereotypic behaviors such as weaving. Provide hay throughout the night (OR = 0.41, 95% CI: 0.16–1.01) were more likely to be very effective management strategy in SA horses compared to VA. Therefore, distraction techniques, as already reported for playing shooting music [70,71], may be useful only for horses with mild anxiety levels. No differences were found in characteristics such as sex, breed and location of the stable. Similarly, in the questionnaire conducted by Lindstedt, the severity of anxiety in different stable locations did not differ significantly [63]. Instead, our results differ from what found in pets: while there is evidence that breed, genetics, age and environment affect the prevalence of noise anxiety in dogs [26,36,37,38,40], in our study only sex came out to be a significant factor, geldings being classified more frequently as “very anxious” compared to mares.

## 4. Conclusions

An online survey was used to investigate UK and US owners’ perception of noise anxiety severity in their horses, the management strategies adopted to reduce anxiety behaviors and their perceived efficacy. The results showed that severe noise anxiety is reported to cause serious welfare consequences, impacting both physiology (e.g., gastrointestinal signs, sweating) and behavior (e.g., running, breaking fence) of the horse. Very anxious horses showed signs of noise reactivity frequently and their reaction did not improve with time (they do not habituate to these stimuli). The most used management strategies consisted of providing hay throughout the night, turning in/out their horse or moving it to a paddock far away; however, most of these techniques were reported to be effective only in the slightly anxious subjects, not in the very anxious ones. Our results confirmed that noise anxiety is a behavioral problem that can lead to important welfare consequences for horses. UK and US owners are quite concerned regarding this issue, particularly the ones whose horses have severe noise anxiety-related problems. New management strategies, including the use of medicinal products, should be considered to reduce behavioral and physiological signs and help horses to cope with noisy events. Since some differences were found in the answers from owners of different countries in terms of noise anxiety’s magnitude and spread, further research is still needed to better understand this behavioral problem.

## Figures and Tables

**Table 1:** Percentage of fear and anxiety behaviors shown during noisy events by horses in each cluster (slightly anxious and very anxious).

Behavior	Frequency	Slightly Anxious (SA) *n* = 181	Very Anxious (VA) *n* = 195
Fence/box walking	Always	5 (3%)	54 (28%)
	Sometimes	117 (65%)	127 (65%)
	Never	52 (29%)	10 (5%)
	Don’t know	7 (4%)	4 (2%)
Running	Always Sometimes Never Don’t know	5 (3%) 121 (67%) 49 (27%) 6 (3%)	43 (22%) 137 (70%) 12 (6%) 3 (2%)
Appetite loss	Always Sometimes Never Don’t know	2 (1%) 56 (31%) 114 (63%) 9 (5%)	23 (12%) 117 (60%) 49 (25%) 6 (3%)
Diarrhea	Always Sometimes Never Don’t know	1 (1%) 41 (23%) 133 (73%) 6 (3%)	16 (8%) 106 (54%) 68 (35%) 5 (3%)
Breaking fence	Always Sometimes Never Don’t know	0 (0%) 23 (13%) 155 (86%) 3 (2%)	4 (2%) 74 (38%) 110 (56%) 7 (4%)
Weaving	Always Sometimes Never Don’t know	2 (1%) 46 (25%) 124 (69%) 9 (5%)	18 (9%) 87 (45%) 83 (43%) 7 (4%)
Bucking	Always Sometimes Never Don’t know	2 (1%) 84 (46%) 94 (52%) 1 (1%)	24 (12%) 119 (61%) 46 (24%) 6 (3%)
Sweating	Always Sometimes Never Don’t know	1 (1%) 107 (59%) 70 (39%) 3 (2%)	38 (19%) 150 (77%) 7 (4%) 0 (0%)
Fever	Always Sometimes Never Don’t know	0 (0%) 3 (2%) 166 (92%) 12 (7%)	3 (2%) 24 (12%) 128 (66%) 40 (21%)
Trembling	Always Sometimes Never Don’t know	1 (1%) 71 (36%) 105 (54%) 4 (2%)	30 (15%) 128 (66%) 32 (16%) 5 (3%)
Vocalization	Always Sometimes Never Don’t know	3 (2%) 92 (47%) 79 (41%) 7 (4%)	32 (16%) 129 (66%) 27 (14%) 7 (4%)

**Table 2:** Summary of binary logistic regression analysis. Odds ratios for the association between various independent variables and cluster horse anxiety level (slightly anxious vs. very anxious). Numbers in bold distinguish the different variables and the statistically significant results.

Category	β	OR	OR 95% CI		Sign.
**Sex** (ref: Mare)					
Gelding	0.398	1.49	0.77	2.86	0.23
**Breed** (ref: Hotblood)					
Coldblood	−0.637	0.53	0.22	1.30	0.17
Warmblood	−0.777	0.46	0.20	1.04	0.06
**Definine the location of the stable** (ref: Urban)					
Rural (surrounded by farms)	0.942	2.57	0.53	12.46	0.24
Rural village on a main road	−20.101	0.00	0.00		1.00
Semi-rural (adjacent to an urban area)	0.847	2.33	0.46	11.96	0.31
**How long does anxious behavior usually last?** (ref: Until the next day or longer)					
The duration of the noisy event	−2.281	0.10	0.01	1.22	0.07
Up to 2 h after the noisy event ended	−1.141	0.32	0.08	1.31	0.11
I don’t know	0.346	1.41	0.32	6.27	0.65
**At what age did your horse start showing these signs?** (ref: <1 year of age)					
1–3 years	0.342	1.41	0.28	7.02	0.68
4–9 years	0.742	2.10	0.47	9.41	0.33
≥10 years	−0.005	1.00	0.17	5.70	1.00
Don’t know	0.202	1.22	0.29	5.15	0.78
**Has your horse’s noise reactivity changed over time?** (ref: Worse with time)					
Better with time	−1.198	0.30	0.09	1.05	0.06
No, it’s the same	−0.259	0.77	0.23	2.64	0.68
**How often does your horse show signs of noise reactivity?** (ref: Once a week)					
Once a month	−0.203	0.82	0.38	1.76	0.61
Less frequently	1.501	4.49	0.22	93.15	0.33
Never	0.115	1.12	0.42	2.99	0.82
**Has the horse injured itself as a result of the reactions caused by the noisy event?** (ref: yes)					
No	−1.406	0.25	0.08	0.75	**0.01**
**In your opinion, which are your horse’s three most alarming behaviors related to noise reactivity?** (ref: Yes)					
Fence/box/stall walking (No)	−0.224	0.80	0.38	1.70	0.56
Breaking through fences (No)	−1.747	0.17	0.06	0.48	**0.00**
Bucking/rearing (No)	−0.934	0.39	0.18	0.87	**0.02**
Decreased appetite (No)	−0.078	0.93	0.32	2.71	0.89
Diarrhea/constipation/colic (No)	−1.459	0.23	0.10	0.56	**0.00**
Fever (No)	1.013	2.75	0.34	22.08	0.34
Running (No)	−0.947	0.39	0.18	0.83	**0.02**
Sweating (No)	−0.684	0.51	0.23	1.10	0.08
Trembling (No)	−0.779	0.46	0.21	1.00	0.05
Vocalisation (No)	−0.386	0.68	0.27	1.70	0.41
Weaving or other stereotypic behaviour (No)	−1.093	0.34	0.12	0.92	**0.03**
**How effective “move the horse to a paddock away from the event” had been?** (ref: Not effective)					0.53
Haven’t tried	−0.342	0.71	0.29	1.75	0.46
Somewhat effective	0.064	1.07	0.45	2.52	0.89
Very effective	0.373	1.45	0.49	4.34	0.50
**How effective “turn in /turn out the horse” had been?** (ref: Not effective)					0.14
Haven’t tried	−1.321	0.27	0.08	0.86	**0.03**
Somewhat effective	−0.134	0.87	0.42	1.84	0.72
Very effective	−0.44	0.64	0.23	1.79	0.40
**How effective “provide hay through out the night” had been?** (ref: Not effective)					0.07
Haven’t tried	−1.972	0.14	0.02	0.82	**0.03**
Somewhat effective	−0.339	0.71	0.34	1.48	0.36
Very effective	−0.904	0.41	0.16	1.01	**0.05**

## Data Availability

The data that support the findings of this study are available on request from the corresponding author E.D.C.

## Supplementary Materials

The following supporting information can be downloaded at: https://www.mdpi.com/article/10.3390/ani12101319/s1, Table S1: Survey questions, Table S2: Owners and Horse populations main characteristics.
