# Supplementary material for: The Impact of Noise Anxiety on Behavior and Welfare of Horses from UK and US Owner’s Perspective
Source: Animals (Basel). 2022 May 21;12(10):1319. doi: 10.3390/ani12101319 (PMC9138043; doi:10.3390/ani12101319)
Supplement: Supplementary file 1 [file animals-12-01319-s001.zip › animals-1661996-supplementary.pdf]

Table S1. Survey questions.

Part	Question	Possible answer
1	Do you own a horse?	No/Yes, 1 horse/Yes, 2 horses/ Yes, 3 or more horses
1	What is your experience with horses?	<2 years/2 – 5 years/6 – 20 years/>20 years
1	Has your horse ever shown any unusual behaviour (e.g. restlessness, sweating, decreased appetite) during noisy events such as firework displays?	Yes/No[1]
2	Age of the horse	<1 year old/1 - 5 years old/6 - 15 years old/>15 years old
2	Sex of the horse	Mare/Stallion/Gelding
2	Breed of the horse	Coldblood/Hotblood/Warmblood
2	Main purpose of your horse	Leisure/Riding /Breeding/Driving/Western/Retired
2	Definine the location of the stable	Rural (surrounded by farms)/Semi-rural (adjacent to an urban area)/Urban (within a town/urban environment)
2	Which of the following behaviors has your horse shown during noisy events?<br>-Fence/box walking<br>-Running<br>-Decreased appetite<br>-Diarrhea/constipa-tion/colic<br>-Breaking through fences<br>-Weaving or other stereotypic behaviour<br>-Bucking/rearing<br>-Sweating<br>-Fever<br>-Trembling<br>-Vocalization	Always/Sometimes/Never/Don't know
2	How long does anxious behavior usually last?	Duration noise event/Up to 2 hours after/Until the next day/I don't know
2	Has the horse injured itself as a result of the reactions caused by the noisy event?	Yes/No
2	How anxious would you rate your horse during noisy events?	Not anxious/Somewhat anxious/Very anxious-terrified
2	Does somebody go and check on the horses during noisy events such as firework displays?	Yes/No
2	In your opinion, which are your horse's three most alarming behaviors related to noise reactivity?<br>-Fence/box walking<br>-Breaking through fences<br>-Bucking/rearing<br>-Decreased appetite<br>-Diarrhea/constipation/colic<br>-Fence/stall walking<br>-Fever<br>-Running<br>-Sweating	Yes/No

	-Trembling<br>-Vocalization<br>-Weaving or other stereotypic behaviour	
2	At what age did your horse start showing these signs?	<1 year/1-3 years/4-9 years/≥10 years/I don't know
2	Has your horse's noise reactivity changed over time?	Better/It's the same/Worse
2	How often does your horse show signs of noise reactivity?	Once a week/Once a month/Less frequently/Never
2	Please indicate if you have tried the following management strategies for your horse during noisy events such as firework displays in previous years and how effective the management strategy was<br>-Move the horse to a paddock away from the event<br>-Turn in / turn out the horse<br>-Move the horse off the property<br>-Sedate the horse (prescription mecidines)<br>-Use over-the-counter products (pheromones, herbal calmers, etc.)<br>-Use ear plugs/cover for the horse<br>-Cover windows<br>-Play music/radio in the barn<br>-Provide hay through out the night	Very effective/Somewhat effective/Not effective/Haven't tried
2	If there was a new oral prescription product for noise reactivity available, would you be interested in using it?	No/Likely no/Likely yes/Yes

[1] Only respondents answering "Yes" could access to the Part 2 of the questionnaire.

Table S2. Owners and Horse populations main characteristics**.**

	**UK owners (N=1220)**	**US owners (N=616)**
How many horses they own		
None	34 (2.8%)	17 (2.8%)
1 horse	678 (55.6%)	250 (41.3%)
2 horses	294 (24.1%)	149 (24.6%)
3 or more horses	214 (17.5%)	189 (31.2%)
How many years of experience with horses they have		
< 2 years	62 (5.1%9	47 (7.8%)
2-5 years	128 (10.5%)	108 (17.9%)
6-20 years	476 (39.0%	278 (46.0%)
> 20 years	554 (45.4%)	172 (28.4%)
How many horses has ever shown any unusual behavior in relation to loud noises		
Yes	275 (22.4%)	134 (22.0%)
No	952 (77.6%)	474 (78.0%)
Horses' age		
< 1 year old	1 (0.4%)	0 (0%)
1-5 years old	25 (9.2%)	13 (9.7%)

6-15 years old	166 (60.8%)	76 (56.7%)
> 15 years old	81 (29.7%)	45 (33.6%)
Horses' sex		
Mare	107 (39.2%)	57 (42.5%)
Stallion	1 (0.4%)	0 (0%)
Gelding	165 (60.4%)	77 (57.5%)
Horses' main purpose		
Riding	174 (64.0%)	39 (29.6%)
Endurance	4 (1.5%)	2 (1.5%)
Western	4 (1.5%(	20 (15.2%)
Trekking	27 (9.9%)	38 (28.8%)
Harness racing	0 (0%)	0 (0%)
Breeding	2 (0.7%)	1 (0.8%)
Other	61 (22.4%)	32 (24.2%)
